# Multilevel Airway Obstruction Phenotypes in Adult OSA

**DOI:** 10.1002/oto2.21

**Published:** 2023-06-23

**Authors:** Bartholomew Bacak, Lee Porterfield, Sveta Karelsky

**Affiliations:** ^1^ University of Rochester Medical Center Rochester New York USA

**Keywords:** drug‐induced sleep endoscopy, obstructive sleep apnea, sleep surgery

## Abstract

**Objective:**

To describe multilevel phenotypes of airway obstruction identified on drug‐induced sleep endoscopy (DISE) in adults.

**Study Design:**

Retrospective chart review.

**Setting:**

Tertiary care center.

**Methods:**

Video recordings of DISE on adult patients were retrospectively scored. A cross‐correlation matrix was created to detect significant correlations between DISE findings at anatomical subsites. Three multilevel phenotypes resulted from the matrix: complete collapse at the tongue base with complete collapse at the epiglottis (T2‐E2), complete circumferential obstruction at the velum with complete lateral pharyngeal wall collapse at the oropharynx (V2C‐O2LPW), and incomplete collapse at the velum with complete collapse due to tonsillar hypertrophy (V0/1‐O2T). The mean difference (MD) and 95% confidence interval (CI) were calculated for demographic and polysomnogram metrics of each phenotype compared to all other subjects.

**Results:**

Phenotype 1 (T2‐E2) (n = 88) had older ages (MD 5.784 years, CI [1.992, 9.576]), lower body mass index (BMI) (MD –1.666 kg/m^2^, CI [02.570, −0.762]), and smaller neck circumferences (MD *–*0.448 in., CI [−9.14, −0.009]) than the other phenotypes. Phenotype 2 (V2C‐O2LPW) (n = 25) had higher BMIs (MD 2.813 kg/m^2^, CI [1.362, 4.263]), higher neck circumference (MD 0.714 in., CI [0.004, 1.424]), and higher apnea‐hypopnea index (MD 8.252, CI [0.463, 16.041]). Phenotype 3 (V0/1‐O2T) (n = 20) had younger ages (MD –17.697, CI [−25.215, −11.179]).

**Conclusion:**

Three distinct multilevel phenotypes of obstruction were identified on DISE, suggesting different anatomic subsites collapse in a nonrandom pattern. The phenotypes appear to represent distinct patient groups and their identification may have implications in terms of pathophysiology and treatment modalities.

Obstructive sleep apnea (OSA) is a common condition with implications for individual health as well as societal disease burden. OSA frequently causes sleep disruption resulting in poor sleep quality, increased daytime sleepiness, and lower quality of life.^1^ OSA is also a common contributor to hypertension and other cardiovascular diseases as well as the pathogenesis of cerebrovascular accidents, as it contributes to hypoxia, activation of the sympathetic nervous system, and endothelial dysfunction.^2^

Positive airway pressure (PAP) therapy has been the gold standard of treatment for moderate to severe OSA for decades. PAP has been shown to improve objective findings of OSA on polysomnography as well as quality of life scores.^3^ However, adherence to PAP therapy is poor and nonadherence rates are approximately 34% to 45%.^4^, ^5^, ^6^ PAP intolerance may be caused by claustrophobia, mask discomfort and air leaks, disruptive noise, restricted movement, and/or failure to achieve adequate sleep with treatment.

The surgical management of OSA has recently gained in popularity and efficacy. Although individual practices vary, many sleep surgeons rely on drug‐induced sleep (sedation) endoscopy (DISE) to identify the anatomical location(s) and morphology of upper airway obstruction to guide surgical management. DISE aims to pharmacologically approximate human sleep and subsequently characterize dynamic collapse of the upper airway during visualization with flexible laryngoscopy. Preoperative DISE can change surgical decision‐making and has been linked to increased surgical success.^7^ The advent of hypoglossal nerve stimulation (HNS) has in particular brought DISE and its interpretation to the forefront of OSA treatment.^8^, ^9^

The field exploring the relationship of DISE findings to clinical care is evolving. Adult OSA patients with lower body mass index (BMI, kg/m^2^) have been found to be more likely to have an obstruction at the tongue base and epiglottis compared to patients with higher BMI.^10^, ^11^ Complete circumferential collapse at the level of the velum, on the other hand, is associated with higher BMI and apnea‐hypopnea index (AHI). Complete lateral oropharyngeal wall collapse is also emerging as a clinically significant pattern associated with more challenging patient habitus and disease severity^12^ and potentially less favorable outcomes of surgical treatment.^8^, ^13^ At the level of the oropharynx, obstruction due to muscular lateral wall collapse has been minimally differentiated in the literature from that caused by palatine tonsillar hypertrophy in terms of patient demographics or clinical significance.

We describe multilevel obstruction patterns visualized on DISE and hypothesize that individual DISE findings of collapse at the velum, oropharynx, tongue base, and epiglottis (VOTE)^14^ may be grouped into phenotypes representing general organizing principles of the adult OSA airway. The concept of such multilevel airway phenotypes has not previously been explored and their identification has potential implications for further study and treatment. We also expand on prior work relating to distinct obstruction morphology of the lateral pharyngeal wall.

## Methods

### Study Design

A retrospective chart review of all consecutive adults with OSA who underwent DISE with dexmedetomidine sedation was conducted at the University of Rochester Medical Center between 2016 and 2020. Exclusion criteria included BMI over 35 kg/m^2^ and use of any other sedative agents. The study was granted Institutional Review Board exemption by the University of Rochester Research Subject Review Board.

### Patient Information

All patients had polysomnograms at a sleep center and/or a home sleep test prior to DISE. Sleep study results, including the AHI, oxygen desaturation index (ODI), and oxygen nadir, were recorded. Sleep study data closest to the time of DISE without intervening airway surgery were used. Demographic factors, neck circumference, BMI, tonsil size, medical conditions, and type and date of any sleep‐related surgeries were also recorded. The American Academy of Sleep Medicine's definition of hypopnea as an event with greater than or equal to 3% oxygen desaturation was used to measure AHI.^15^

### DISE

DISE procedures were performed in operating rooms at the University of Rochester by the principal investigator, an attending otolaryngologist with extensive DISE experience. DISE was performed in a semidark operating room with the patient lying in the supine position on a hospital bed with a pillow and blanket to approximate normal sleeping conditions. Standardized intravenous dexmedetomidine infusion was performed at a rate of 1 mcg/kg over 10 minutes, then after 10 minutes switched to a maintenance rate of 1 mcg/kg/h. The patient was observed for transition to unconsciousness, snoring, or other evidence of upper airway obstruction, nonresponse to verbal and tactile stimuli, as well as oxygen desaturation. The Covidien bispectral index monitoring system (Covidien‐Medtronic) was used to assess sedation.^16^ Endoscopic video examination was performed and only portions corresponding to appropriate sedation were recorded and saved (D‐Scope Systems). We performed secondary assessments with jaw thrust for select patients to identify if they would benefit from mandibular advancement. However, we did not consistently perform assessments with jaw thrust and therefore excluded this data from the present study. De novo scoring was performed for all recordings at the time of this study rather than using the score captured at the time of DISE to reduce the effect of patient and disease factors on scoring. The previously described VOTE scoring system^14^ was utilized with the following modifiers to describe the pattern of collapse at the oropharynx: tonsillar tissue (T) and muscular lateral pharyngeal wall (LPW). The distinction between lateral collapse due to tonsillar tissue versus muscular lateral pharyngeal wall was previously described by our group.^11^

### Identification of Phenotypes and Statistical Analysis

A 2‐dimensional cross‐correlation matrix was generated with the grade and configuration of airway obstruction. We retrospectively classified subjects by grade of obstruction as either “incomplete” or “complete” obstruction. Therefore, subjects with grade 0, that is, no obstruction, and grade 1, that is, incomplete obstruction, using the traditional VOTE scoring system,^14^ were classified as “incomplete.” Subjects with grade 2 obstruction were classified as “complete.” The decision to separate the degrees of obstruction into “incomplete” (grade 0/1) and “complete” (grade 2) is consistent with Kezirian et al's finding that the interrater reliability for the presence of obstruction on DISE was higher than for the degree of obstruction.^17^ Configurations of complete obstruction at the velum (V) and oropharynx (O) were circumferential (2C) versus anteroposterior (2AP) and lateral muscular pharyngeal wall (2LPW) versus static obstruction by palatine tonsil tissue (2T), respectively. *χ* ^2^ tests were performed to identify significant correlations and the effect size was measured with Cramer's *V*. The resulting significant correlations yielded 3 main airway obstruction phenotypes: complete collapse at the tongue base with complete collapse at the epiglottis (T2‐E2) or phenotype 1; complete circumferential obstruction at the velum with 2LPW obstruction at the oropharynx (V2C‐O2LPW) or phenotype 2; no complete collapse at velum with complete tonsillar collapse (2T) at the oropharynx (V0/1‐O2T) or phenotype 3. Age, BMI, neck circumference, AHI, ODI, and oxygen nadir for the phenotypes were compared using the Student's *t* test with *α* = .05. Individual phenotypes were first compared to all other subjects. Interphenotype analysis was also performed using the same metrics. The effect size was quantified with Cohen's *d*, where the effect size is commonly interpreted as small, medium, and large for values of 0.2, 0.5, and 0.8, respectively.^18^ Statistical analysis was performed using JASP v0.16.^19^

## Results

There were no incidents of harm or adverse events in this study.

### Demographics

N = 263 subjects were identified; 31.6% (n = 83) were female and 68.4% (n = 180) were male. The demographics of the study population are summarized in Table 1.

**Table 1:** Demographics of the Study Population.

	Mean	Standard deviation	Range
Age (y)	50.901	14.960	18, 84
BMI (kg/m^2^)	27.889	3.594	15.7, 34.9
Neck circumference (in.)	16.073	1.495	12.0, 20.0
AHI	29.549	18.934	0.7, 102.5
ODI	24.299	17.285	0.4, 87.9
O_2_ nadir (%)	81.303	8.112	35, 95

Abbreviations: AHI, apnea‐hypopnea index; BMI, body mass index; ODI, oxygen desaturation index.

### Subgroup Analysis

Subjects categorized into a phenotype (n = 147) were found to be distributed into 3 statistically significant phenotypes based on the degree and configuration of collapse at anatomical subsites. Seven subjects were grouped into two phenotypes due to grade 2 obstruction at all anatomic levels and were therefore excluded from phenotypes for further analysis; three had 2T obstruction and four had 2LPW obstruction. The sample sizes and relative statistical strengths of the respective phenotypes according to Cramer's *V* test are shown in Table 2. Tables 3, 4, 5 show the comparison of each phenotype to the remainder of the study population. Phenotype 1 was the largest of the 3 phenotypes at 33.5% of the cohort and demonstrated a significant inverse correlation with grade 2C velum obstruction (*χ* ^2^ = 17.505, *p* < .001, Cramer's *V* = 0.258) as well as 2LPW oropharynx obstruction (*χ* ^2^ = 12.080, *p* < .001, Cramer's *V* = 0.214).

**Table 2:** Correlation and Effect Size for Phenotype Classification.

	n	*χ* ^2^	*p*	Cramer's *V*
Phenotype 1: T2‐E2	88	139.539	<.001	0.728
Phenotype 2: V2C‐O2LPW	25	31.331	<.001	0.345
Phenotype 3: V0/1‐O2T	20	8.939	.003	0.184

A 2 × 2 contingency table (df = 1) was constructed for each phenotype and *χ* ^2^ and Cramer's *V* statistics were then calculated.

**Table 3:** Phenotype 1: T2‐E2.

	Phenotype 1: T2‐E2	All others	Mean Difference	95% Confidence Interval	Cohen's d
Age (years)	54.750	48.966	5.784	1.992, 9.576	0.393
BMI (kg/m^2^)	26.781	28.447	−1.666	−2.570, −0.762	−0.474
Neck Circumference (in.)	15.759	16.232	−0.448	−0.914, −0.009	−0.319
AHI	29.416	29.615	−0.200	−5.081, 50.81	−0.011
ODI	25.402	23.801	1.600	−3.539, 6.739	0.092
O2 Nadir (%)	80.945	81.483	−0.538	−2.638, 1.562	−0.066

Comparison with the Student's *t* test between the phenotype and all subjects not in that phenotype. Values in shaded boxes demonstrated statistical significance (*p* < .05).

Abbreviations: AHI, apnea‐hypopnea index; BMI, body mass index; ODI, oxygen desaturation index.

**Table 4:** Phenotype 2: V2C‐O2LPW.

	Phenotype 2: V2C‐O2LPW	All others	Mean Difference	95% Confidence Interval	Cohen's d
Age (years)	50.480	50.945	−0.465	−6.670, 5.740	−0.031
BMI (kg/m^2^)	30.435	27.622	2.813	1.362, 4.263	0.803
Neck Circumference (in.)	16.711	15.997	0.714	0.004, 1.424	0.482
AHI	37.016	28.764	8.252	0.463, 16.041	0.439
ODI	26.889	24.035	2.854	−5.362, 11.071	0.165
O2 Nadir (%)	79.280	81.518	2.238	−5.593, 1.117	−0.276

Comparison with the Student's *t* test between the phenotype and all subjects not in that phenotype. Values in shaded boxes demonstrated statistical significance (*p* < .05).

Abbreviations: AHI, apnea‐hypopnea index; BMI, body mass index; ODI, oxygen desaturation index.

**Table 5:** Phenotype 3: V0/1‐O2T.

	Phenotype 3: V0/1‐OT	All others	Mean Difference	95% Confidence Interval	Cohen's d
Age (years)	34.550	52.247	−17.697	−24.215, −11.179	−1.244
BMI (kg/m^2^)	29.128	27.788	1.340	−0.301, 2.981	0.374
Neck Circumference (in.)	15.821	16.094	−0.273	−1.095, 0.550	−0.182
AHI	34.195	29.166	5.029	−3.639, 13.697	0.266
ODI	29.529	23.828	5.701	−2.913, 14.316	0.330
O2 Nadir (%)	80.800	81.345	−0.545	−4.269, 3.178	−0.067

Comparison with the Student's *t* test between the phenotype and all subjects not in that phenotype. Values in shaded boxes demonstrated statistical significance (*p* < .05).

Abbreviations: AHI, apnea‐hypopnea index; BMI, body mass index; ODI, oxygen desaturation index.

**Figure 1:**
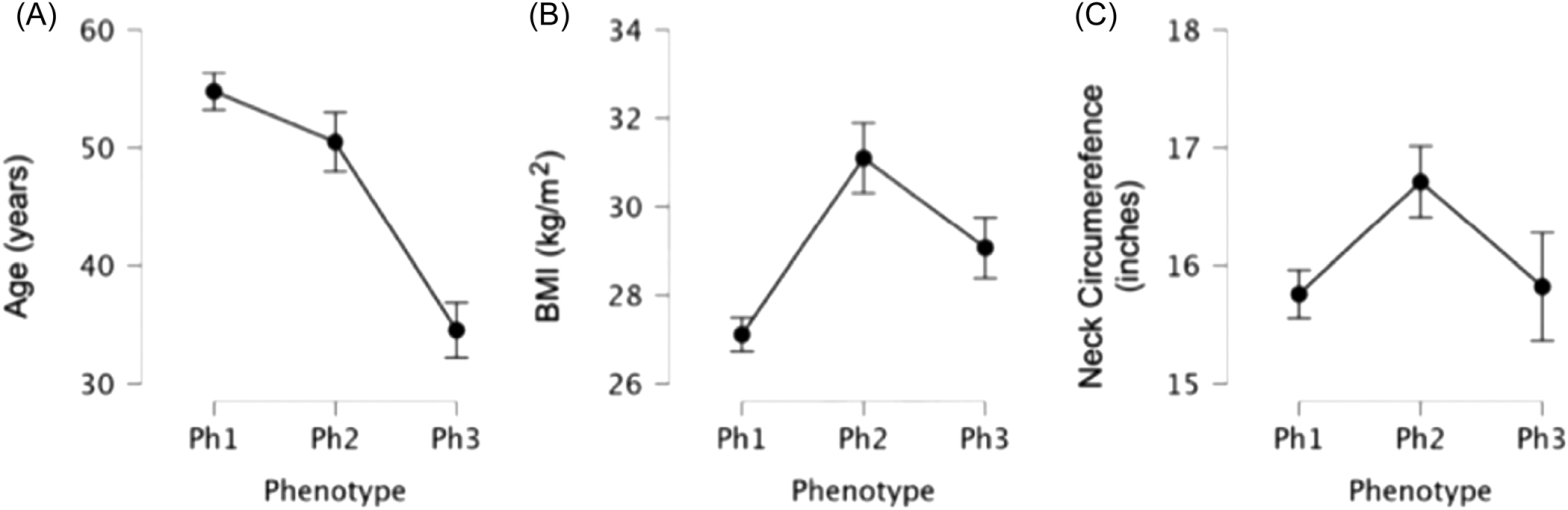
Comparison of (A) age, (B) BMI, and (C) neck circumference across phenotypes (Ph1‐3). Mean values are depicted with error bars indicating standard deviation. BMI, body mass index.

Interphenotype analysis was performed with the same methodology described above for the analysis of individual phenotypes versus all others. The mean (*M*) BMI of phenotype 1 (T2‐E2) was *M* = 26.781 kg/m^2^, which was significantly less than that of phenotype 2 (V2C‐O2LPW) *M* = 30.435 kg/m^2^ (confidence interval, CI [−5.135, −2.170]) and phenotype 3 (V0/1‐O2T) at *M* = 29.128 kg/m^2^ (CI [−4.000, −0.694]). The neck circumference of phenotype 1 (T2‐E2) at *M* = 15.759 in. was significantly smaller than that of phenotype 2 (V2C‐O2LPW) at *M* = 16.711 in. (CI [−1.746, −0.157]). The mean age of phenotype 3 (V0/1‐O2T) was *M* = 34.550 years, which was significantly younger than that of phenotype 2 (V2C‐O2LPW) at *M* = 50.480 years (CI [−22.977, −8.883]) and phenotype 1 (T2‐E2) at *M* = 54.750 years (CI [−27.106, −13.294]). All other interphenotype comparisons failed to achieve statistical significance. The interphenotype analysis is illustrated in Figure 1.

Subjects that were not classified into a phenotype were compared to all those classified into one or more of the phenotypes across the same metrics listed in Tables 3, 4, 5, and no significant differences were detected.

## Discussion

We describe multilevel, statistically significant phenotypes of airway obstruction as seen on DISE in adult OSA patients. This finding suggests that airway levels described in the VOTE classification do not obstruct independently of each other, but rather do so in potentially meaningful combinations. Three minimally overlapping phenotypes emerge and appear to represent distinct patient groups. Further, when complete lateral oropharyngeal obstruction is divided into two configurations, 2LPW and obstruction by the palatine tonsils 2T,^11^ the two groups fall into distinct phenotypes. While disease phenotypes in the global pathogenesis of OSA have previously been discussed, little attention has been paid to the idea of phenotypic groupings of airway anatomy in OSA.^20^, ^21^, ^22^

**Figure 2:**
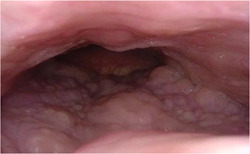
Representative DISE image of phenotype 1 demonstrating tongue base and epiglottic obstruction without obstruction at the velum or oropharynx. The velum is seen at the bottom of the figure.

The statistically strongest and most common phenotype identified in this study was phenotype 1, comprised of significant obstruction by the base of the tongue and epiglottis. The typical DISE appearance of phenotype 1 is seen in Figure 2. The identification of a pattern in which significant tongue base and epiglottic obstruction co‐occur is unsurprising: the tongue base and epiglottis are closely related anatomically and the tongue in many cases displaces the epiglottis to contribute to its collapse. It is rather the absence of concurrent 2C velum and 2LPW oropharyngeal obstruction that represents the clinical significance of this airway phenotype. Base of tongue obstruction in the absence of 2C velum obstruction or complete lateral pharyngeal wall obstruction typically responds well to treatment with HNS (8). Additional characteristics of phenotype 1 patients may also make them favorable surgical candidates. Phenotype 1 patients have a lower BMI than both the rest of the cohort and patients in the other phenotypes, and 59% of patients with BMI in the normal range (18.5‐24.9 kg/m^2^) in our cohort were in this phenotype. The neck circumference of the phenotype 1 patients is significantly lower than the rest of the cohort. When separated by sex, the neck circumference of phenotype 1 subjects was *M* = 14.12 in., standard deviation [SD] = 1.44 for females and *M* = 16.52 in.,  and SD = 0.92 for males; these results suggest that phenotype 1 subjects have similar neck circumferences to the general population.^23^ Therefore, neither obesity nor extraluminal airway compression by the soft tissues of the neck appears to be a significant culprit of OSA in this patient group; hypotonia of the tongue and epiglottic collapse appear to be sufficient. This is consistent with prior studies suggesting that obstruction by the tongue base is associated with lower BMI.^10^, ^11^ A relevant animal study found that in non‐overweight mice, temporary deactivation of hypoglossal motor neurons was sufficient to induce obstructive sleep‐disordered breathing.^24^ Lastly, phenotype 1 patients are older compared to the rest of the cohort, and it has previously been suggested that increased age contributes to the lower tone of the pharyngeal dilators, such as the genioglossus, represented by tongue base collapse.^25^, ^26^, ^27^ Notably, phenotype 1 does not reflect patients with isolated epiglottic obstruction, a relatively rare but important finding on DISE.

**Figure 3:**
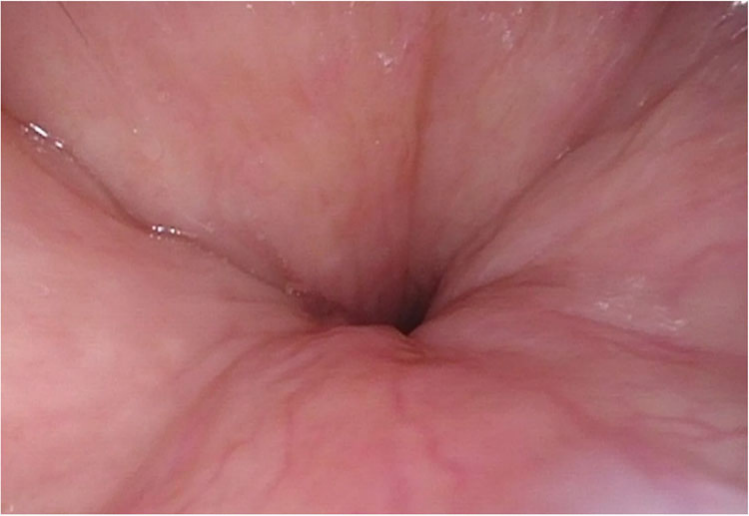
Complete circumferential collapse at the velum as seen in phenotype 2. A near‐perfect circle is seen at the genu of the palate on inspiration on DISE.

**Figure 4:**
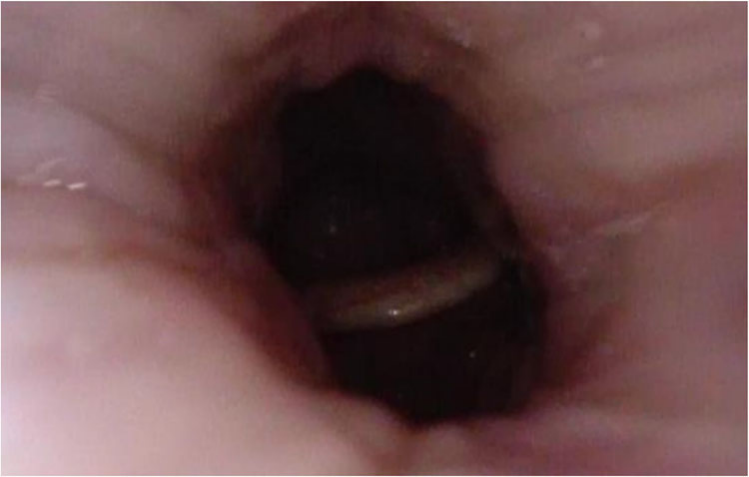
Dynamic lateral muscular pharyngeal wall collapse at the level of the oropharynx during early inspiration. The image is captured in mid‐collapse.

Unlike phenotype 1, phenotype 2 represents the combination of two clinically unfavorable DISE findings: grade 2 or complete concentric collapse at the velum (Figure 3) and grade 2 lateral pharyngeal wall collapse (Figure 4). Both have emerged as patterns that portend greater OSA severity, higher BMI, and greater difficulty with PAP‐alternative treatment, most notably resulting in the exclusion of the former from treatment with HNS by the US Food and Drug Administration. Their significant co‐occurrence may represent a globally unfavorable airway obstruction pattern and mark certain adult OSA patients as difficult to treat with PAP alternatives. In agreement with prior findings for the airway levels involved, phenotype 2 was found to have significantly greater BMI, AHI, and neck circumference compared to the remainder of the cohort. Further studies are needed as to the physiologic basis for this pattern, possibly involving measurement of the tone of relevant airway musculature in this group as has previously been done in OSA patients.^25^, ^26^ Consistent association with higher BMI also introduces the question of whether these findings would improve with weight loss.

PAP‐alternative treatment modalities for obstruction patterns comprising phenotype 2 have been explored both as standalone surgical therapy in the form of expansion sphincter pharyngoplasty,^28^ and also in the interest of converting a circumferential palate to a more favorable AP obstruction configuration utilizing a modified uvulopalatopharyngoplasty.^29^ The emerging treatment approach of conversion of the complete concentric collapse of the velum to an AP configuration followed by HNS is of interest.

The discussion of phenotype 2 must address the immediate anatomic proximity of the velum and oropharynx, and therefore the contribution of lateral wall oropharyngeal obstruction to a circular airway appearance at the velum on DISE. Although it is often not possible to view and interpret the velum and oropharynx independently of each other on DISE, we scored the velum configuration at the level of the genu of the palate to attempt to overcome this problem.

**Figure 5:**
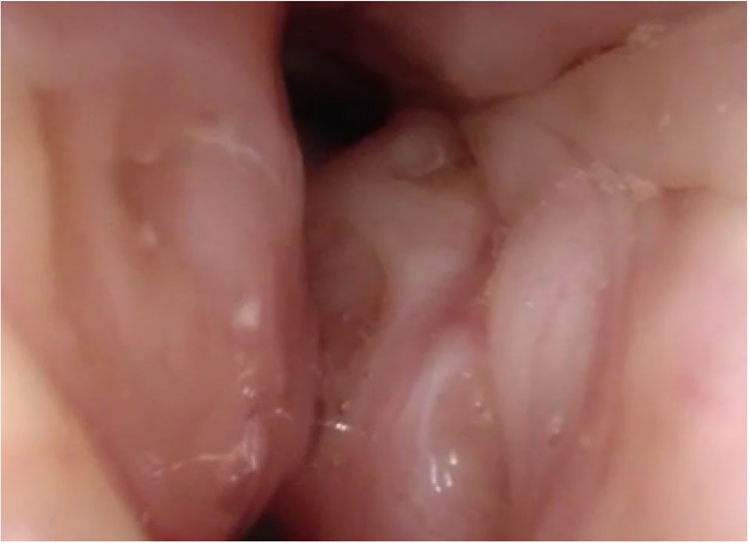
Airway obstruction at the oropharynx by bulky palatine tonsil tissue, as seen in phenotype 3.

Further differentiation of significant oropharyngeal collapse appears desirable due to the morphologically variable obstruction encountered at this level. It has become apparent in our clinical practice that lateral oropharyngeal obstruction is divided into the true dynamic collapse of the muscular lateral pharyngeal wall (Figure 4) and more static obstruction by exophytic palatine tonsil tissue (Figure 5), which has previously been described. Individuals in the latter category who also had an absence of significant obstruction at the velum comprised one of the statistically significant phenotypes in our correlation matrix. This group, termed phenotype 3, was significantly younger compared to the rest of the cohort and each of the other phenotypes. Although there was a relatively small number of patients in this phenotype, which may affect the statistical significance, the findings are consistent with prior work on 2T patients. The lack of concurrent obstruction at the velum may represent stenting of the airway by the bulky exophytic tonsils as well as true mutual exclusivity of obstruction. Among patients with 2T obstruction at the oropharynx, 65% had documented tonsil size on awake transoral examination, and of those, only 53% were classified as tonsil size 3+ or larger. 2T obstruction on DISE is, therefore, not synonymous with marked palatine tonsil enlargement on awake transoral examination, and DISE is likely still valuable for these patients. We suggest that an addition to the current VOTE scoring system should be considered to account for these distinct oropharyngeal obstruction configurations. The most clinically relevant question about phenotype 3 is whether treatment with tonsillectomy is more likely to be effective in this group than in other adults with OSA, and outcomes studies for tonsillectomy in this group are needed. It is also not known whether additional levels of obstruction on DISE would develop without the airway stenting effect of large palatine tonsils.

The three identified phenotypes did not encompass all subjects, an important consideration when interpreting this study.  Forty‐five percent of subjects (n = 119) were not included in a phenotype. These patients were not significantly different from those in a phenotype in terms of demographic or sleep study metrics. Many nonphenotype subjects did not have grade 2 obstruction at any airway level, explaining their exclusion given the current study design. It is likely that the separation of grade 0 and grade 1 collapse would identify a different phenotype distribution and include more of the study population in a phenotype. Similarly, we expect that additional phenotypes may emerge if a 3‐ or 4‐dimensional correlation matrix were employed. However, this should be carried out with a larger patient cohort.

There are additional limitations to this study. DISE is an inherently subjective methodology, compounded in this case by the impossibility of blinding oneself to obstruction levels not being evaluated during multilevel airway visualization. The physiological causes of the identified phenotypes may include neurologic, muscular, and craniofacial factors including the position of the mandible during sleep, which were not analyzed in our study. In this study, we considered both home‐ and lab‐based sleep studies; the former tends to underestimate OSA severity. Lastly, mandibular positioning and improvement in airway patency during DISE after a jaw thrust maneuver were not recorded in this study and deserve further study.

## Conclusion

We identify multilevel patterns of upper airway obstruction, or airway phenotypes, in adults with OSA. These phenotypes likely have clinical significance based on what is already known about their component subsites. Further evaluation of airway phenotypes, their physiologic basis, and any relationship to broader OSA phenotypes may be valuable for the diagnosis and treatment of OSA.

## Author Contributions

**Bartholomew Bacak**, design, analysis, interpretation, and drafting; **Lee Porterfield**, data gathering, analysis, and drafting; **Sveta Karelsky**, design, interpretation, and drafting.

## Disclosures

### Competing interests

None.

### Funding source

None.
